# The Changing Presentation of Choledochal Cyst Disease: An Incidental Diagnosis

**DOI:** 10.1155/2009/103739

**Published:** 2009-10-19

**Authors:** Rajeev Dhupar, Brian Gulack, David A. Geller, J. Wallis Marsh, T. Clark Gamblin

**Affiliations:** ^1^Liver Cancer Center University of Pittsburgh Medical Center, 3459 Fifth Avenue, Pittsburgh, PA 15213, USA; ^2^The University of Pittsburgh School of Medicine, Scaife Hall, Pittsburgh, PA 15261, USA

## Abstract

*Background*. Choledochal cysts are uncommon biliary lesions. Due to the evolution of imaging and laparoscopic surgery, we sought to describe our last 3 years experience with the presentation and management of choledochal cysts in adults. *Methods*. A retrospective review of a prospectively established database of adults who were managed for primary choledochal cyst disease between 2005 and 2008 was performed. *Results*. Between 8/2005 and 8/2008, 14 adults were managed for primary choledochal cyst disease. The average age was 41 years (range 17–86) and 79% were female. Presentations included biliary sepsis (3), pancreatitis (2), abdominal pain (3), or painless jaundice (1). Three patients had the cyst found during laparoscopic cholecystectomy, and two had an incidental finding after CT scan for an unrelated issue. The length of stay for those who had the cyst removed was 7.8 days (range 5–11). There were no operative or post-operative complications. *Conclusions*. Over the last 3 years 36% of our patients with choledochal cysts presented after incidental finding, either during a laparoscopic operation or after a CT scan for an unrelated problem. Increasing utilization of laparoscopy and CT scan for abdominal complaints has lead to a change in the pattern of presentation.

## 1. Background

Choledochal cysts (CDCs) are uncommon, yet well documented congenital dilations of the intra- and/or extra-hepatic biliary tree that can carry significant morbidity if not recognized and treated early [1]. Traditionally, there are five varieties of CDCs that are classified according to their location in the biliary tree by the Todani modification of the Alonso-Lej classification [2]. Classically, this disease has been described to have a higher incidence of diagnosis during the first decade of life as well as a predominance in females of 3-4:1 [3–5]. Additionally, there is a much higher prevalence of CDCs in southeast Asian countries, with an overall incidence of 1:1000 compared with 1:100 000–150 000 in Western countries. An unexplained phenomena, however, is that Asian immigrants to Western countries do not carry the high incidence present in their native countries [1, 4, 6]. CDCs are associated with the congenital biliary anomaly pancreaticobiliary maljunction (PBM) as well as a higher lifetime risk for the development of cholangitis, acute pancreatitis, and cholangiocarcinoma; therefore, immediate excision or liver transplantation is recommended [1, 4, 7–11]. However, because of the relatively rare presentation of this disease in western countries, much of the current understanding of the presentation and management is derived from large Asian series [3].

Recently, Edil et al. reported the largest single institution western experience with CDC management and concluded that surgical intervention greatly reduces or perhaps eliminates the risk of cholangiocarcinoma [9]. Additionally, while confirming the need for excision of CDCs to prevent associated morbidity, this and multiple other reports have suggested that the most common presentation of this disease in adults is rarely the classically described “triad” of right upper quadrant pain, jaundice, and palpable abdominal mass. Instead, these reports suggest that adults are most likely to present with abdominal pain, are much less likely to have jaundice, and rarely if ever have an abdominal mass [1, 9, 12, 13]. While these studies cover a large number of patients, they also span a long period of time, some up to 30 years [1, 8, 9, 12]. While valuable in helping us understand the long-term outcomes associated with surgical management, they do not describe how the presentation of CDCs has changed with the evolution of medicine and technology. Over the last few decades there have been advances in the field of radiology that significantly impact how many diseases are diagnosed and ultimately managed. We therefore undertook a study of our recent experience with CDCs in adults in order to determine if the evolution of medical technology has changed the presentation, management, or short term outcomes of CDC disease from what is described in reported series that span a much broader time period. Here we report 14 patients over 3 years that were diagnosed with CDCs at a single institution and describe the presentation, management, and outcomes of these patients.

## 2. Methods

Approval for this study was obtained from the Institutional Review Board. A search for “choledochal cyst” was performed for all patients receiving an operation at the UPMC Liver Cancer Canter between 2005 and 2008. We analyzed patient demographics, presentation, type of cyst, pathology, operation, and complications. A series of 14 patients with the above criteria was found and all were included in the study.

## 3. Results

### 3.1. Patient Demographics and Presentation

Fourteen adults were managed for primary CDC disease over a three year period. The average age was 41 years (range 17–86) and 79% were female. The median age for the women was 24 years (range 17–62), while the median age for the men was 68 years (range 65–86). All three men presented with biliary sepsis, while the women presented with either pancreatitis (2), abdominal pain (3), painless jaundice (1), incidental finding during visual inspection or cholangiogram during laparoscopic cholecystectomy (3), or incidental finding after CT scan for an unrelated issue (2) (Tables 1 and 2).

### 3.2. Diagnosis, Management and Outcomes

Fifty-eight percent of the patients had an ERCP, 29% had MRCP only, and no patients were diagnosed or further evaluated by ultrasound. Nine patients had a Todani type I cyst, 1 had a type II cyst, 1 had a type III cyst, 1 had a type IV cyst, and 2 had a type V cyst (Table 1). The presence of pancreaticobiliar maljunction was not discerned in these patients, as all would undergo operative intervention that would excise the CDC and undergo biliary diversion. All patients with type I cysts had excision with a Roux-en-Y hepaticojejunostomy. The patient with a type III cyst had a Whipple procedure because the patient had persistent symptoms of pain and nausea despite endoscopic sphincterotomy, and the cyst involved the pancreatic duct extending into the pancreatic head. The patients with types IV and V cysts had hepatic lobectomies appropriate for the extent of disease with or without Roux-en-Y reconstruction. The patient with a type II cyst did not have it removed because of decompensated liver cirrhosis and was found after laparoscopic cholecystectomy to have biopsy proven hepatocellular carcinoma (Table 1). The average length of stay for those who had the cyst removed was 7.8 days (range 5–11). There were no operative or postoperative complications. All pathology has revealed benign disease, and all patients have had an uneventful followup.

## 4. Discussion

Early detection and treatment of CDCs is an important factor in the overall lifetime occurrence of cholangiocarcinoma. The lifetime risk of CDC associated cholangiocarcinoma is as high as 26% in some studies, and importantly, the rate of occurrence increases with age [1, 8, 13–15]. Patients discovered in their twenties have only a 2.3% risk of concomitant malignancy, but this increases to 75% in patients with CDCs discovered in their eighties [1, 8, 13–15]. Once found, the current treatment of choice is surgical excision, as it is well documented to lead to a decrease in rate of malignancy from 16% to less than 1% [7, 9, 14].

CDCs are also associated with another rare congenital biliary anomaly, pancreaticobiliary malformation (PBM). PBM is the extraduodenal fusion of the pancreatic duct and common bile duct beyond the sphincter of Oddi, and while found in a high proportion of patients with type I CDCs, they are thought to be distinct embryologic and clinical entities [10]. In our series, while 64% of the patients had type I CDCs, it was not specifically noted if PBM was present as well. While the ultimate management of these patients may not have been different, the association of PBM with CDC merits study, as this relationship provides a potential pathogenesis for the causes of the morbidity that underlies CDCs, including cholangiocarcinoma [10, 16].

Treatment of CDCs prior to the onset of symptoms is recommended, as the benefit of reducing the risk of cholangiocarcinoma outweighs the risk of surgery prior to the development of symptoms [12]. Recently, Lipsett et al. reported that while the ratio of incidence of CDCs in adults versus children was 1 : 1 three decades ago, it has risen to almost 4 : 1 [12, 13]. When these adults present, it is usually with the chief complaint of abdominal pain, and rarely do adults with CDCs have jaundice or an abdominal mass [1, 9, 12, 13]. However, the symptoms are not pathognomonic and the classically described triad of abdominal pain, jaundice, and palpable mass is more the exception than the rule [5, 6, 9, 17].

Historically, management of CDCs consisted of various drainage operations, but high complication rates and retained threat of malignancy have led to the use of excision or transplantation as first-line therapy [5, 18]. The preferred surgical operation is cyst removal and Roux-en-y hepaticojejunostomy, and depending on cyst type (Todani type IV, V), further intervention may be necessary [2, 4, 12, 18]. All of our patients, whether symptomatic or asymptomatic, were treated with surgical excision. If intrahepatic involvement was present, hepatic lobectomy was performed. Fortunately, although CDCs associated cholangiocarcinoma are generally found in 11% of cases, it was not found in any of our patients [1, 8, 13–15]. This may lie partly in the fact that the risk of CDC associated cholangiocarcinoma increases with age, and the average age of our patients was 41 years. Only 3 of our patients were over age 65, and one had associated HCC. However, it is likely that with increased numbers of patients the rate of cholangiocarcinoma will meet what is found in the broader population. Unfortunately, due to the short followup in this study of patients recently diagnosed and treated for CDC, it is not yet possible to evaluate and compare long-term outcomes from nontraditional discovery. Generally, our followup in patients without malignancy consists of yearly LFTs and evaluation for new symptoms, but not yearly imaging.

As medicine and medical technology have evolved, so has the presentation of CDCs at our institution. In the last decade there have been significant improvements in the quality of medical imaging technology as well as greater access and utilization of more sophisticated imaging devices such as CT scanners. Additionally, the more widespread use of minimally invasive surgery has made it possible for procedures to be diagnostic as well as therapeutic and has obviated the need to undertake an unplanned large operation and instead refer patients to appropriate specialists. In our series, while the majority of patients presented symptomatically, 36% seen at our center over three years presented with asymptomatic CDCs. Two patients had CDCs found by CT scan, one for left upper quadrant pain and one for pelvic pain from a ruptured ovarian cyst. Three were found during laparoscopic cholecystectomy for acalculous or chronic cholecystitis. These patients were not found to have choledochal lithiasis and did not have the choledochal cyst identified on ultrasound examination during their initial work-up. It can be presumed that the CDCs were missed on the ultrasounds, however this cannot be confirmed. One was a type I cyst seen on inspection during the cholecystectomy, one was a type IV cyst seen on cholangiogram, and one was a type V cyst seen on cholangiogram (Table 2). For those patients that did present with symptoms, most presented with abdominal pain (21%), and few presented with jaundice (7%) or abdominal masses (0%), which is in agreement with findings from the previous reports [1, 9, 12, 13]. Additionally, the characteristics of our patients match what is commonly reported in western adult populations, with a female to male ratio of roughly 4 : 1 and a majority being Todani Type I (64%) [1, 9, 12, 13].

Choledochal cysts, an uncommon malformation of the biliary tree, can lead to significant morbidity and mortality if not treated promptly [4, 9]. Traditionally regarded as a disease of childhood, over the last two decades many reports are finding a higher percentage of patients with CDCs presenting as adults [6, 12, 13]. In addition, we report that a significant percentage of CDCs may now present without abdominal pain, but rather as an incidental finding on CT scan, cholangiogram, or laparoscopic surgery. At our institution over the last 3 years, more than 1/3 of the CDCs have presented through nontraditional routes of diagnosis. While the most common presentation for CDCs is still abdominal pain, centers may soon recognize that a significant portion of patients with this disease present incidentally. This may impact not only on the future risk of developing cholangiocarcinoma, but diagnosis and treatment prior to onset of symptoms may allow for an elective resection that avoids postoperative complications.

## Figures and Tables

**Table 1:** Demographic information of all patients including age, gender, presentation, type of cyst, operation, and presence of malignancy on initial pathology.

Patient	Age	Sex	Cyst type (Todani)	Presentation	Operation	Malignancy at operation
1	24	F	I	Incidental	Roux-en-y hepatico J^a^	No
2	24	F	IV	Incidental	R lobectomy, hepatico J	No
3	49	F	I	Abdominal pain	Roux-en-y-hepatico J	No
4	41	F	V	Incidental	R lobectomy	No
5	62	F	I	Incidental	Roux-en-y-hepatico J	No
6	19	F	I	Abdominal pain	Roux-en-y-hepatico J	No
7	17	F	I	Incidental	Roux-en-y hepatico J	No
8	41	F	I	Jaundice	Roux-en-y-hepatico J	No
9	22	F	I	Abdominal pain/ pancreatitis	Roux-en-y-hepatico J	No
10	17	F	I	Pancreatitis	Roux-en-y-hepatico J	No
11	44	F	III	Abdominal pain (post cholecystectomy)	Whipple	No
12	68	M	II	Biliary sepsis	Laparoscopic cholecystectomy	Yes (HCC)^b^
13	86	M	V	Biliary sepsis	L lobectomy, removal impacted stone	No
14	65	M	I	Biliary sepsis	Roux-en-y-hepatico J	No

a: hepaticojejunostomy; b: hepatocellular carcinoma.

**Table 2:** Presentation of choledochal cyst, number of patients with each presentation, and the percentage with each presentation.

Presentation	*n*	%
Biliary Sepsis	3	21
Pancreatitis	2	14
Abdominal Pain	3	21
Jaundice	1	7
Incidental Presentation	5	36
* * *-CT scan *	2	14
* * *-Cholangiogram *	2	14
* * *-visual inspection during elective operation *	1	7
